# Fertility-Sparing Surgery for Non-Epithelial Ovarian Malignancies: Ten-Year Retrospective Study of Oncological and Reproductive Outcomes

**DOI:** 10.3390/cancers17081304

**Published:** 2025-04-12

**Authors:** Ivana Likic Ladjevic, Jelena Dotlic, Katarina Stefanovic, Branislav Milosevic, Aleksandra Beleslin, Olga Mihaljevic, Jovan Bila, Ivana Vukovic, Milos Radojevic, Zoran Vilendecic

**Affiliations:** 1 Clinic for Obstetrics and Gynecology, University Clinical Centre of Serbia, Dr Koste Todorovica 26, 11000 Belgrade, Serbiadrenadot@gmail.com (J.D.);; 2 Medical Faculty, University of Belgrade, Dr Subotica 8, 11000 Belgrade, Serbia

**Keywords:** non-epithelial ovarian cancers, recurrence, survival, pregnancy, oncological outcomes, reproductive outcomes, fertility-sparing surgery, personalized treatment

## Abstract

Background/Objectives: Due to the rarity and histological heterogeneity of non-epithelial ovarian cancers (NEOCs), monitoring their reproductive and oncological outcomes is challenging. Therefore, this study aimed to investigate the oncological and reproductive outcomes of patients with NEOCs treated with fertility-sparing surgery over the past 10 years at our tertiary referral university clinic. Methods: This retrospective study included all the NEOC patients diagnosed and treated with fertility-sparing surgery from 2010 to 2019. The patient demographic and clinical characteristics; data regarding the treatment andthe clinical, laboratory, and imaging findings during follow-up; and disease recurrences were recorded. In this study, the recurrence-free survival and the overall survival were the oncological outcomes. The reproductive outcomes were assessed as attempting and achieving pregnancy. Results: This study included 39 patients. The most frequent NEOCs were granulosa cell tumors (53.8%). The majority of the tumors were in the IA or IC1 stage. The initial therapy was generally a unilateral salpingo-oophorectomy (30.8%). Adjuvant chemotherapy was received by 48.7% of the patients. An NEOC recurrence was registered in 25.6% of the patients, mostly during the first two postoperative years. The recurrence-free survival was 76.92%. A regression analysis showed that amore advanced stage of NEOC was the most important predictor of disease recurrence. The overall survival rate was 87.2%, with a mean time to an adverse outcome of 23.01 +/−10.68 months. The regression analysis showed that better survival depended mostly on not having disease recurrence. After treatment, ten patients tried to conceive and seven succeeded. All the children were in good condition upon birth. Conclusions: Fertility-sparing treatment for NEOCs was proven as a safe and successful option in terms of both oncological and reproductive outcomes.

## Simple Summary

Non-epithelial ovarian cancers (NEOCs) are a histologically diverse tumor group, and therefore, their diagnostics andthe monitoring of patient outcomes are difficult. This study aimed to investigate the oncological and reproductive outcomes of patients with NEOCs treated with fertility-sparing surgery over the past 10 years at our clinic. In our study, we confirmed that fertility-sparing treatment for NEOCs is a safe and successful option in terms of both the oncological and reproductive outcomes. Moreover, we determined that, out of all the tumor and treatment characteristics of the different patients, a more advanced stage of NEOC was the most important predictor of disease recurrence, while better survival depended mostly on not having disease recurrence. After treatment for an NEOC, the pregnancy rate reached 70% at our clinic. Our findings can help practitioners personalize the diagnosis and treatment of NEOCs. Further research on the risk factors for NEOCs using larger samples is planned.

## 1. Introduction

Non-epithelial ovarian cancers (NEOCs) encompassa wide spectrum of rare ovarian malignancies, such as malignant germ cell tumors (GCTs), sexcord–stromal tumors (SCSTs), ovarian sarcomas, and small-cell carcinomas of the ovary [1,2]. NEOCs account for only 3% to 10% of all ovarian malignancies, with a yearly incidence of between 2.1 and 3.7 per 1,000,000 women [3]. The early symptoms and signs of NEOCs generally include a sense of pressure or pain in the pelvis orabdomen as well asirregular menstruation. Although most NEOCs present in similar ways, due to their rarity, histological heterogeneity, and variableage distribution, it is challenging to monitor the incidence of these conditions, treat them, and measure the reproductive and oncological outcomes [1,4].

GCTs originate from the primordial germ cell and mostly occur in younger women of reproductive age, but can also be found in postmenopausal women. SCSTs arise from the sex cord and ovarian stroma and are mainly diagnosed in older peri- and postmenopausal women, although recently, they have more often been registered in younger, premenopausal women [1]. Ovarian carcinosarcomas, also known as mixed malignant Mullerian tumors, are a rare, biphasic, and histological subtype composed of an epithelial part as well as a sarcomatous component. Each element contributes to the development of the malignancy to a different extent. The prognosis for this condition is adverse in most cases, and most patients relapse within one year after completing the initial treatment [5]. Several risk factors for NEOCs have been established in previous research, such as a family history of ovarian or other gynecological malignanciesor associated genetic syndromes or mutations, obesity, smoking, a sedentary lifestyle, and endometriosis for clear-cell carcinoma [2,3].

Diagnoses are based on abdominal and pelvic ultrasound (US) and magnetic resonance imaging (MRI) results, as well as chest X-rays for distant metastases. From a biochemical perspective, although all of the markers for NEOCs are nonspecific, different markers can be useful in different cases, as NEOCs are a diverse group of tumors. Therefore, a full blood count should be performed, the liver and renal function should be checked, and the serum levels of cancer antigen 125 (CA 125), beta human chorionic gonadotropin (β HCG), α-foetoprotein (AFP), inhibin B, and anti-Mullerian hormone (AMH) should be examined [1,2,3].

While, for epithelial ovarian cancers, radical surgery is the therapy of choice, the treatment for NEOCs is based on the patient’s age, their previous parity, and the tumor’s histological type and stage. Conservative fertility-sparing surgery (FSS) is the standard initial treatment for all NEOCs, while platinum-based chemotherapy is applied as an adjuvant treatment. FSS can be performed in most patients with NEOCs, as these tumors are generally diagnosed in the early stages, as opposed to epithelial ovarian cancers, which are usually diagnosed late. The ideal conservative treatment for advanced stages remains a subject of debate. It is believed that, even in such cases (especially with GCTs), FSS can be performed (as the cure rates from this approach are high), but it should be accompanied by chemotherapy. Nevertheless, due to the diversity of patient characteristics and circumstances and of the NEOCs themselves, personalized treatment is optimal whenever possible [1,2,6,7].

Although the overall prognosis of NEOCs is favorable (high survival rate), the main issue among reproductive-aged patients is the impact of the surgery, especially if it involves chemotherapy, on their residual ovarian function and fertility potential [7,8,9]. Given that NEOCs, especially GCTs, are often diagnosed in girls and young women who generally have not yet finished reproducing, preserving fertility without compromising oncological management is a crucial goal.

The unique challenges associated with preserving fertility in NEOC patients can be explained by the rarity and histological heterogeneity of NEOCs, which have made it difficult to develop standardized guidelines for fertility-sparing treatment. Consequently, many patients and even some physicians are unaware of the options for preserving reproductive function prior to malignancy treatment. On the one hand, conservative management enables further reproduction; on the other hand, it leaves open the risks of cancer persistence, progression, and recurrence, or even adverse outcomes. In particular, the recurrence rates are high when only a cystectomy is performed. However, these facts do not significantly affect the overall survival. Therefore, each young patient should be evaluated individually, and conservative treatment should be proposed whenever possible [2,10].

In the existing literature, the conception rates after fertility-sparing NEOC treatment vary from 16% to 59%, while the pregnancy rates range from 67% to 100%. However, most studies have been performed with small samples; consequently, the existing data on the reproductive and obstetrical outcomes remain insufficient [11,12,13].

The aim of this study was to investigate the oncological and reproductive outcomes of patients with non-epithelial ovarian tumors who underwent fertility-sparing surgery over 10 years at our tertiary referral university clinic. As the oncological outcomes, we considered the patient’s overall health status during follow-up, including NEOC recurrence and the overall survival, while the main reproductive outcome was the successful achievement of pregnancy.

## 2. Materials and Methods

This retrospective study included all patients diagnosed with NEOCs and treated with fertility-sparing surgery at the Clinic for Gynecology and Obstetrics, University Clinical Center of Serbia, over a 10-year period (2010–2019). The management of oncological patients at our clinic, a tertiary referral center that specializes in preserving the fertility of gynecologic oncology patients, is regulated and supervised by a multidisciplinary team operating as part of the Council for Gynecological Oncology and the Council for Cancer and Human Reproduction.

The inclusion criteria were as follows: patients must have been between 18 and 45 years of age, had an adequately surgically staged ovarian malignancy, underwent fertility-sparing surgery as the initial treatment, and had regular follow-ups after surgery for at least 12 months according to current protocols. The exclusion criterion was concurrent malignancies or comorbidities that might impact fertility, such as a myoma or polycystic ovary syndrome (PCOS). This criterion was set to minimize confounding effects when testing the obstetric outcomes. Patients with missing data and those lost during follow-up were also excluded from the study. This study was approved by the Ethical Review Board of the University Clinical Center of Serbia (2023, No. 576/4). All the subjects involved in the study granted their informed consent to participate.

The patients’ clinical and demographic characteristics were obtained from their medical records at the time of their diagnosis and treatment. Data on each patient’s oncologicaloutcome (overall health status during follow-up) and reproductive outcome (success in achieving pregnancy) were obtained from the medical records of their regular check-ups.

The preoperative assessment for all patients included calculating their body mass index (BMI); conducting a chest X-ray and imaging of the pelvis and abdomen (US with a follicular count and MRI); taking their family, personal, medical, and gynecological history; conducting a thorough gynecological examination, including a colposcopy and Papanicolaou testing; and completing laboratory testing, including conducting biochemical analyses and examining the blood count, coagulation factors, and tumor markers CA 125, human epididymis protein 4 (HE4), AFP, and carcinoembryonic antigen (CEA), as well asAMH (to assess the ovarian reserve).

The data collected on the surgical procedure included the chosen type of surgery (FSS or radical), the indications (staging and/or definite treatment), the approach (laparotomy or laparoscopy), the number (a one- or two-step procedure), and the time of each procedure for each patient.

FSS was chosen as the treatment for young patients and patients with a strong desire to continue to reproduce. The decision was based on a preoperative assessment that indicated a low-stage disease and having favorable oncological selection criteria for ovarian preservation according to current guidelines. Postoperatively, the final choice of therapeutic approach was made in accordance with the histological findings [4,14,15]. FSS was defined as any surgery that preserved the uterus and at least part of one ovary, including a unilateral cystectomy, unilateral oophorectomy, unilateral salpingo-oophorectomy (USOE), or bilateral cystectomy. The surgical staging included a USOE, pelvic lymphadenectomy, partial omentectomy, and peritoneal washing for cytological examination. The staging could be complete or incomplete. Radical surgery (a hysterectomy with a bilateral salpingo-oophorectomy) was performed as a second-step treatment if indicated. Upon surgical treatment, all the tumors were histologically analyzed, categorized according to the World Health Organization (WHO 2014)’s classification system, and staged in accordance with the International Federation of Gynecology and Obstetrics’ staging classification [2,13]. The findings were used to determine whether adjuvant chemotherapy was indicated (a protocol based on administering bleomycin, etoposide, and platinum–BEP).

Follow-up was performed according to the current standard protocols and included a regular clinical examination, laboratory testing, and an US or MRI in cases of suspicious findings or increased tumor markers [4,15]. We checked up on each patient every three months during the first and second years, every six months until the end of the fifth postoperative year, and then annually. The follow-up duration for each patient was noted.

In cases of recurrence, the localization and time of recurrence, as well as the subsequent treatment, were documented. The recurrence-free survival (RFS, measured as the months from the initial surgical NEOC resection to the date of the recurrence diagnosis) and the overall survival (OS, measured as the months from the surgical NEOC resection to the date of an adverse outcome, i.e., death from any cause) were also determined for every patient.

Finally, we noted all patients who attempted to become pregnant. Success in becoming pregnant (yes/no) was assessed as the main reproductive outcome of the study. Women who achieved pregnancy were closely monitored at our clinic according to the established protocols for high-risk pregnancies. Moreover, we registered the mode of conception (spontaneous or via assisted reproduction—ART), the pregnancy outcome (miscarriage, preterm delivery, or term delivery), and the delivery mode (vaginal or caesarean section).

### Statistical Analysis

The data were analyzed using the SPSS software (version 20.0, SPSS, Inc., Chicago, IL, USA). Differences in the characteristics of the patients, the tumors, and the performed treatments were assessed using a chi square (χ^2^) test. Associations of the investigated parameters with the oncological and obstetric outcomes were examined using Spearman’s correlation (ρ). A regression analysis was applied to assess which patient, tumor, and treatment characteristics could impact NEOC recurrence and patient survival. The overall and recurrence-free survivals were investigated using the Kaplan–Meier method, with a log-rank test for time-to-event outcome comparisons. The cut-off for statistical significance was set at *p* <0.05.

## 3. Results

This study included 39 patients with NEOCs who were, on average, 26.87 ± 6.95 years of age (range: 17–41; median: 27 years). The patients with an SCST were significantly older at the time of their diagnosis than the patients with a GCT. The investigated patients had up to two pregnancies before their NEOC, but most were nulliparous (66.7%). Descriptive data of the investigated patients are presented in Table 1.

In total, 22 (56.4%) patients were diagnosed with SCSTs and 17 (43.6%) were diagnosed with GCTs (*p* = 0.423). The single most frequent NEOC was a granulosa cell tumor (53.8%), out of which 43.6% were adult-type and 10.3% were juvenile-type tumors. In the SCST group, we observed one case of a Sertoli–Leydig cell tumor. Dysgerminomas were the most common subtype of GCTs, occurring in 20.5% of cases, followed by immature teratomas (12.8%) and yolk-sac tumors (10.3%). At the time of the diagnosis, the majority of the tumors were in the IA or IC1 stage. All the investigated NEOCs were unilateral. The histological subtypes and stages of the investigated NEOCs are presented in Table 2.

A unilateral salpingo-oophorectomy as the initial fertility-sparing treatment was the therapy of choice for 30.8% of the patients (Table 2). Staging was completed as a one-step procedure in the majority of cases, while a second-step surgery was indicated in 28.2% of the investigated NEOC patients. In our sample, five patients had only a USOE without further staging. Out of the patients who had staging, 11 were completely staged, while the others were not. Cytology testing and an omental biopsy were performed for all the staged patients, a biopsy of the contralateral ovary and peritoneum was performed for about 40% of the staged patients, and a lymphadenectomy was performed for only six patients. Nevertheless, there were no positive findings for the lymph nodes, the biopsy of the other ovary, the omentum, or the peritoneum, or for the cytology testing. A laparotomy was the preferred approach for the initial surgery in 79.5% of the patients and for all (100%) cases of second-step surgeries. An initial laparoscopic surgery was performed in eight patients. After the surgical treatment, adjuvant chemotherapy was indicated in 48.7% of the patients. They received from two up to six cycles of BEP chemotherapy (on average, 1.56 ± 1.94).

### 3.1. OncologicalOutcomes—Recurrence and Survival

The mean follow-up time was 62.92 ± 45.65 months (range:9–120 months) and 56.4% of the patients were followed for more than five years.

**Figure 1:**
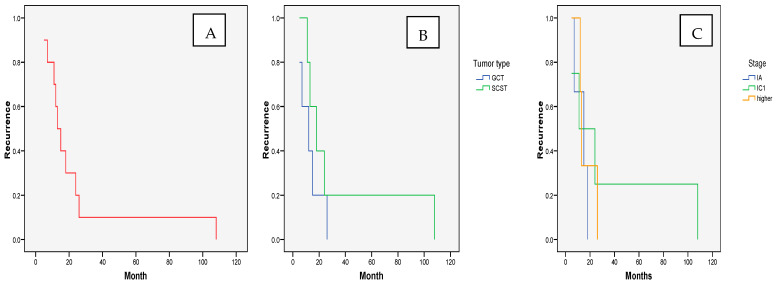
Time to NEOC recurrence after conservative therapy: (**A**). overall and according to (**B**). tumor type and (**C**). stage.

The recurrence of NEOCs was registered in a quarter of the patients (Table 2). Although, among recurrent diseases, all tumor types were registered, the highest recurrence rates were registered for Sertoli–Leydig (100%) and granulosa cell (50%) tumors. Recurrence was generally diagnosed in the first two postoperative years (mean time to recurrence: 23.91 ± 30.29; median: 11; range: 5–108 months), although one of our patients had the recurrence of an immature teratoma in the ninth year of follow-up. The recurrence-free survival at the end of the first postoperative year was 92.31%. At the end of the third year, it was 76.92% and remained unchanged almost until the end of the follow-up period. The recurrence-free survival was similar for both GCTs and SCSTs (*p* = 0.317). There were also no significant differences in the duration of recurrence-free survival with regards to the NEOC stage (*p* = 0.758) (Figure 1).

Having a recurrent NEOC was correlated with a more advanced tumor stage at the time of diagnosis (*p* = 0.036) and not with the performance ofa second-step surgery (*p* = 0.021). There were no significant associations of patient, tumor, or therapy characteristics with the time to disease recurrence or its localization. The obtained regression analysis model (*p* = 0.011) showed that amore advanced stage of NEOC at the time of diagnosis was the most important factor that could impact disease recurrence (Table 3).

Surgical radicalization due to NEOC recurrence was performed in four patients: one with a yolk sack tumor, two with granulosa cell tumors, and one with a Sertoli–Leidig cell tumor. Moreover, nine patients received additional BEP chemotherapy.

During the follow-up period, a lethal outcome was observed in five patients with NEOCs, giving an overall survival rate of 87.2% for NEOCs (Table 2). Adverse outcomes were registered for two patients with granulosa cell tumors: one with a yolk sack tumor, one with an immature teratoma, and one with a dysgerminoma. Although the SCST group had somewhat better survival compared to the GCT group, the difference was not significant (*p* = 0.434).

**Figure 2:**
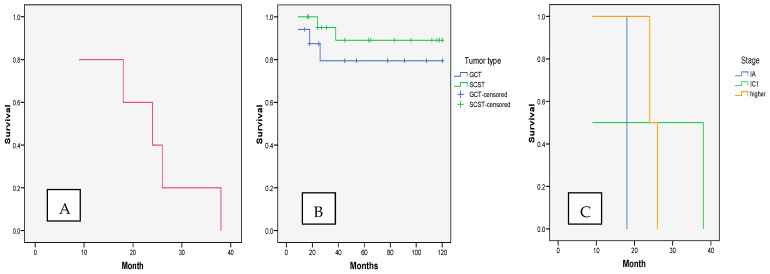
Time to adverse outcome in NEOC patients: (**A**). overall survival and according to (**B**). tumor type and (**C**). stage.

Adverse outcomes occurred mostly between the 20th and 30th postoperative months (mean time to adverse outcome: 23.01 ± 10.68; median: 24; range: 9–38). The duration to an adverse outcome was similar for both GCTs and SCSTs (*p* = 0.441). There were also no significant differences in the duration of recurrence-free survival with regards to the NEOC stage (*p* = 0.622) (Figure 2).

The overall survival of patients with an NEOC was correlated with a less advanced tumor stage at the time of the diagnosis (*p* = 0.038), the lack of a NEOC recurrence (*p* = 0.001), and treatment of the recurrence with chemotherapy (*p* = 0.001). On the contrary, the type of surgery (*p* = 0.557) and the initial administration of chemotherapy was not significantly correlated with the overall survival (*p* = 0.601). The obtained significant regression analysis model (*p* = 0.002) showed that the better survival of patients with an NEOC depended mostly on not having disease recurrence (Table 4).

### 3.2. Reproductive Outcomes

Following the initial treatment, ten (25.6%) of our patients tried to conceive and seven succeeded (pregnancy rate: 70%) (Table 5). There were no significant differences in pregnancy achievement between patients with a GCT and those with an SCST. Pregnancy was achieved in two patients with a dysgerminoma, one with an immature teratoma, and four with a granulosa cell tumor of the adult type. Five patients who achieved pregnancy were in stage IA, while two were in the IC1 stage. The examined patients achieved pregnancy in an interval of 1 to 6 years after their NEOC diagnosis. Only one NEOC patient achieved pregnancy by assisted reproduction (ovarian stimulation and IVF/ET), while the others conceived spontaneously. No miscarriages were registered and the achieved pregnancies were uneventful. The mode of delivery for most patients was a natural birth (71.4%). Two children of the patients with granulosa cell tumors were born preterm, but all the children were live-born singletons in good condition upon birth (Table 5).

Three patients were treated with chemotherapy due to an NEOC prior to pregnancy achievement (two patients 2 years and one 4 years after treatment). Out of the patients with recurrent disease, only one with a dysgerminoma spontaneously achieved pregnancy before the recurrence. No significant correlations of the investigated patient, tumor, or treatment characteristics with pregnancy achievement were found. Term delivery was correlated with having a second-step surgery (*p* = 0.001) and not having positive lymph nodes (*p* = 0.001) or a positive peritoneal biopsy (*p* = 0.001). In the regression analysis, no significant models were obtained for pregnancy achievement or delivery time.

## 4. Discussion

NEOCs are generally diagnosed in younger women of childbearing age, except for granulosa cell tumors, which are found across a wide spectrum of ages, including both pre- and postmenopausal women. The peak incidence of GCTs occurs in women aged 15–19 years, while 70% of GCTs are diagnosed in women younger than 30 years of age [10,16]. On the other hand, there is limited knowledge concerning the diagnosis, natural history, treatment, and outcome of GCTs in postmenopausal patients. Any postmenopausal woman with an ovarian mass and elevated serum AFP levels should be suspected of having a GCT. However, as AFP is not routinely tested in this population, arriving at the correct diagnosis can be challenging. The prognosis for such cases is poor, even among patients in the early stages [17]. Almost all the patients we examined were 20–40 years old (eight were <20 years and two were ≥40 years old). In some investigations, parity has been found to increase the occurrence of GCTs and decrease the occurrence of SCSTs [18,19]. The results of our study did not confirm these findings, as no correlations between parity and NOEC occurrence were found.

Research suggests that the incidences of GCTs and SCSTs vary in different populations. In some studies, malignant GCTs (teratomas and dysgerminomas) have been the most frequently discovered histological subtype (at around 50% of all NEOCs), while in others, SCSTs have been registered more frequently than GCTs [7,20,21]. In our sample, granulosa cell tumors were the most common.

According to the literature, patients with NEOCs have a low recurrence and good long-term survival rates, mostly because approximately 60–80% of both SCSTs and GCTs are diagnosed in the early stages. In a study of pediatric patients, almost 70% of NEOCs were in stage I, even though most tumors had diameters > 10 cm [5,10,16]. In our sample, everyone was in the first stage of the disease, aside from one patient.

Previous studies have revealed that the five-year survival rates for NEOCs (both GCTs and SCSTs) can be as high as 98% [9,19,20]). Studies have found that the overall mortality decreases significantly with each calendar year (significantly in the first two years and more gradually afterwards) [7]. Research indicates that the factors associated with lower rates of overall survival include disease recurrence, a residual tumor, incomplete surgical staging, and a more advanced NEOC stage at the time of the diagnosis. The worst prognosis was found for yolk sac tumors [6,20,22]. In some investigations, the 5-year overall survival rate was around 88% for localized NEOCs, but just 34% for NEOCs with distant metastases [7]. Our study confirms that a less advanced NEOC stage at the time of the diagnosis is correlated with better survival. However, the most important risk factor for patient survival is disease recurrence.

The rates of recurrent NEOCs generally vary between 5% and 25%, but in some populations, they are higher than 80% [9,18,22,23]. Recurrent NEOCs in previous research have been registered 6 to 60 months after the initial diagnosis. Late recurrence is mostly diagnosed in patients with granulosa cell tumors. Recurrence is only high in patients with advanced-stage SCSTs that have a negative impact on the patient’s overall survival (≤70%) [9,19]. The literature indicates that the risk factors for NEOC recurrence include an advanced stage, lymph node involvement, and intraoperative capsule rupture [23]. In our patients, recurrent diseases occurred quite frequently (around 25%) and mostly early (up to 24 months after the diagnosis). Other studies have also identified high recurrence rates, despite an excellent overall survival [24].

Surgery is the treatment of choice for the majority of both primary and recurrent NEOC cases [2,4]. In patients who have finished reproduction, a total hysterectomy with a bilateral salpingo-oophorectomy should be performed. Contrarily, conservative fertility-sparing surgery (i.e., a USOE with complete surgical staging, including cytology as well as peritoneal and omental biopsies) is the initial treatment for patients who are still planning to become pregnant [25]. Some past studies have found similar outcomes (survival and recurrence) in patients treated with both radical and conservative surgery if the patients are correctly matched with the treatment [3,17]. However, in the available literature and guidelines, there are noteworthy disparities regarding lymph node resection, ranging from systematic lymph node dissection to the removal of only macroscopically suspicious nodes. The European Society for Medical Oncology suggests the resection of lymph nodes only for recurrent GCTs and not for SCGTs grossly confined to the ovaries. Contrarily, the National Comprehensive Cancer Network recommends a more expansive approach to lymph node dissection [3,11]. Some authors recommend avoiding an ovarian biopsy, as it may cause adhesions and infertility due to ovarian function failure. Therefore, these authors suggest that a contralateral ovarian biopsy should be performed only when the ovary exhibits macroscopic pathological features [6,11]. In our study, lymph nodes were discovered in six patients, and 15 biopsies of the contralateral ovary tested positive. However, these findings did not significantly affect the patients’ overall survival.

Patients with early-stage disease generally do not require adjuvant therapy and should only be closely monitored and followedup, as adjuvant therapy does not seem to significantly impact the prognosis in such cases. However, recurrence can be more frequent in patients who have not received adjuvant chemotherapy [2,17,26]. Debulking surgery followed by adjuvant chemotherapy is the most effective treatment for all advanced-staged NEOCs. Still, it should be noted that GCTs are significantly more chemo-sensitive than SCSTs, which indicates that different therapeutic approaches are needed for these two tumor groups. Therefore, some authors suggest that adjuvant chemotherapy should be applied for GCTs and recurrent cases of SCSTs [9,17,26]. Previous investigations have shown that fertility-sparing treatment can be safely indicated, even for advanced NEOC stages, as they are very sensitive to chemotherapy. Consequently, some authors have reported that more than 80% of their patients received adjuvant therapy [21].

Adjuvant chemotherapy with bleomycin, etoposide, and cisplatin is the gold standard for ovarian malignancies. It improves the outcomes (survival rates following BEP are over 80% for early-stage and 75% for late-stage disease), but can have numerous adverse effects [15,26]. Therefore, there is a need for novel treatments for GCT recurrence based on genetic and proteomic analyses of cancer tissue. It was recently shown that different types of GCTs involve characteristic expressions of certain biomarkers. Accordingly, treatments targeting the EGFR, PI3K, and c-KIT pathways have been assessed in contemporary investigations. Still, many agents, including everolimus, imatinib, sunitinib, and pazopanib, have shown limited effectiveness, with the reported response rates ranging from 0% to 13% in recurrent GCT cases [27,28]. All of our patients who received chemotherapy were treated using the BEP protocol without any major complications or adverse effects. Moreover, it seemed that chemotherapy did not significantly impact our patients’ reproductive potential, as 30% of the patients treated with BEP successfully achieved a pregnancy. Still, it should be noted that only a few of our patients underwent chemotherapy. Therefore, studies with larger samples are needed to confirm our findings.

The practice of undertaking a secondary cytoreductive surgery for patients with recurrent or progressive GCTs is currently under debate. Second-look surgery should be considered for patients with incompletely resected tumors that contain teratoma elements. In practice, a second resection should generally be limited to patients who have a residual immature teratoma with normal tumor markers after adjuvant chemotherapy or in cases of growing teratoma syndrome [2,29].

The radicalization of surgery after childbearing remains debatable, as most early-stage diseases are cured by the initial treatment, although it is mostly suggested to avoid any chance of recurrence [2,17].

The available literature on pregnancy achievement and the outcomes after conservative fertility-sparing surgery for NEOCs is scarce and heterogeneous. The reported pregnancy rates vary from around 20% to more than 90%, while the live birth rates vary from 65% to 95% [9,10,17]. Most of the women in previous investigations who successfully conceived and delivered had GCTs [7]. The conceptions reported in previous studies were mostly natural, and patients generally had term deliveries. Studies have shown that the patient’s age, the type of surgery, recurrence, and the tumor’s histological subtype have the greatest impact on fertility rates [17]. Notably, receiving adjuvant chemotherapy has not been proven to have any negative effects on fertility or obstetrical outcomes [9]. Moreover, pregnancy and delivery after conservative treatment (surgery and chemotherapy) for an NEOC does not seem to negatively impact the recurrence or mortality rates [12]. We registered a 70% rate of pregnancy after conservative fertility-sparing surgery for NEOCs. All the pregnancies ended in favorable outcomes for the mothers and children.

In women with a low or moderate risk of recurrence, pregnancy should not be delayed following the completion of conservative fertility-sparing surgery for NEOCs. Patients can be advised to become pregnant six months after a negative follow-up [4,15]. Pregnancy can be achieved naturally or via ART, as there is no evidence that the use of ART negatively influences patients’ oncological outcomes. Ovarian stimulation can be performed in patients with favorable prognostic factors (i.e., histological tumor type, hormone sensitivity, cancer stage, and oncological prognosis). Nevertheless, some data indicate that letrozole should be avoided in ovarian hyperstimulation cases to prevent any risk of increased recurrence. To preserve fertility, preservation harvesting and the cryopreservation of oocytes prior to a cytoreductive intervention (bilateral oophorectomy) can be considered. The use of ovarian cortex cryopreservation is still debatable due to the possible risks of malignant reseeding [30]. Our patients achieved a pregnancy 12 to 72 months after conservative treatment for an NEOC, and mostly naturally.

This study has three main clinical implications. The recurrence rates were the highest during the second postoperative year, recurrence was the highest for Sertoli–Leydig and granulosa cell tumors, and neither the recurrence rates nor the overall survival significantly differed by tumor stage. In some studies, recurrence has similarly been quite frequent for granulosa cell tumors, but infrequent for Sertoli–Leydig cell tumors [22]. However, the data also indicate that no differences in the RFS are attributable to the tumor type [22]. Currently, we have no clear explanation for these findings. The further analysis of why specific NEOC subtypes may behave more aggressively could yield valuable clinical insights.

One of this study’s limitations was the relatively small cohort of NEOC patients, which limited the generalizability of our findings. Specifically, our findings should not be generalized to perimenopausal patients, as there were not enough of these women in the investigated sample. In addition, the strict inclusion and exclusion criteria set for this study may have caused some selection bias. Thus, the results should not be generalized to women with multiple significant comorbidities. Moreover, due to the small sample, we were unable to separately examine the patient outcomes according to the NEOC histological subtype. This issue limited the exploration of effect estimates. However, as NEOCs are rare tumors, our sample is comparable to those of other single-center investigations. Although our study was performed at one institution, it is the tertiary referral center for gynecological oncology in our country. Moreover, a 10-year period was covered to optimize the reliability of the data. Another limitation is that the minimal follow-up period was 12 months, which might have prevented the diagnosis of recurrence in some patients. The lack of long-term follow-up for some patients could have prevented some late recurrences from being detected. However, more than half of the examined patients were followedup for longer than five years. Furthermore, even though the regression models were adjusted for multiple covariates, it is always debatable whether all the potential confounding factors were included, so residual confounding cannot be ruled out. Certainly, larger multicentric studies with longer follow-up times are recommended to obtain additional evidence regarding the long-term oncological and reproductive outcomes of NEOC patients.

## 5. Conclusions

Based on our 10 years of experience, fertility-sparing treatment for NEOCs is a provably safe and successful option in terms of both oncological and reproductive outcomes. The overall survival rate in our study was 87.2%, while the recurrence rate was 25.6%. Recurrence appeared mostly in the first two years, and it was most common in patients with granulosa cell tumors. A more advanced tumor stage at the time of diagnosis and the decision not to perform the second-step surgery were associated with NEOC recurrence, while a less advanced tumor stage at the time of diagnosis, a negative peritoneal biopsy, the absence of NEOC recurrence, and the use of chemotherapy to treat the recurrence were associated with better overall survival.

For the quarter of patients who attempted to bear children, the pregnancy rate after conservative fertility-sparing surgery for NEOCs was 70%.All the children were live-born and in good condition at birth. The types of initial treatment and adjuvant chemotherapy chosendid not seem to affect the fertility outcomes.

## 6. Patients

This study included all otherwise-healthy NEOC patients aged 18 to 45 years who were diagnosed and adequately surgically staged, treated with fertility-sparing surgery, and regularly followedup according to current protocols for at least 12 months at our center. According to the guidelines, adequate surgical staging for conservative NEOC therapy represents an intraoperative criterion that incorporates an omentectomy, a contralateral ovarian biopsy, peritoneal washing for a cytology analysis, the careful inspection of all peritoneal surfaces, a biopsy of suspicious areas, and lymph node dissection [4,15,16,29]. In accordance with the study criteria, four patients had to be excluded—one had untreated PCOS, which may have impacted her reproductive outcomes, while three had missing data, as they were lost to follow-up.

## Figures and Tables

**Table 1:** Descriptive data of investigated patients.

Parameters	Whole Sample		GCT		SCST		Between Groups *p*
	Mean	Standard Deviation	Mean	Standard Deviation	Mean	Standard Deviation	
Patient’s age	26.87	6.95	23.88	4.59	29.18	7.65	0.016
Parity before cancer	0.46	0.72	0.35	0.61	0.55	0.81	0.415
Follow-up in months	62.92	45.63	64.00	45.43	77.59	42.31	0.342
Recurrence in months	23.91	30.29	13.01	8.28	34.80	41.23	0.281
Adverse outcome in months	23.01	10.68	17.66	8.50	31.01	9.89	0.203

Legend: GCT—germ cell tumor, SCST—sex cord–stromal tumor.

**Table 2:** Frequency of investigated tumor and treatment parameters and the differences between groups.

Parameters		Whole Sample		GCT		SCST		Between Groups *p*
		Number	Percent	Number	Percent	Number	Percent	
Ovarian tumor histology	dysgerminoma	8	20.5	8	47.1	/	/	/
	teratoma	5	12.8	5	29.4	/	/	
	yolk sack	4	10.3	4	23.5	/	/	
	granulosa—adult	17	43.6	/	/	17	43.6	
	granulosa—juvenile	4	10.3	/	/	4	10.3	
	Sertoli–Leidig	1	2.6	/	/	1	2.6	
Cancer stage	IA	21	53.8	10	58.8	11	50.0	0.636
	IC1	13	33.3	5	29.4	8	36.4	
	IC2	3	7.7	1	5.9	2	9.1	
	IC3	1	2.6	0	0	1	4.5	
	IIIC	1	2.6	1	5.9	0	0	
I Surgery type	UCE	5	12.8	1	5.9	4	18.2	0.828
	USOE	14	35.9	6	35.3	8	36.4	
	staging	20	51.3	10	58.8	10	45.5	
II Surgery	no	28	71.8	13	76.5	15	68.2	0.573
	staging	11	28.2	4	23.5	7	31.8	
I Chemo- therapy	no	20	51.3	7	41.2	13	59.1	0.273
	yes	19	48.7	10	58.8	9	40.9	
Recurrence	no	29	74.4	12	70.6	17	77.3	0.640
	yes	10	25.6	5	29.4	5	22.7	
Recurrence localization	peritoneum	4	40.0	1	20.0	3	60.0	0.154
	lymph nodes	2	20.0	1	20.0	1	20.0	
	ovaries	3	30.0	2	40.0	1	20.0	
	multiple	1	10.0	1	20.0	0	0	
II Chemo- therapy	no	30	76.9	13	76.5	17	77.3	0.954
	yes	9	23.1	4	23.5	5	22.7	
Final radicalization	no	35	89.7	16	94.1	19	86.4	0.734
	yes	4	10.3	1	5.9	3	13.6	
Surviving follow-up	no	5	12.8	3	17.6	2	9.1	0.434
	yes	34	87.2	14	82.4	20	90.9	

Legend: GCT—germ cell tumor, SCST—sex cord–stromal tumor, UCE—unilateral cystectomy, USOE—unilateral salpingo-oophorectomy.

**Table 3:** Regression model for NEOC recurrence after conservative therapy.

Parameters	B Coefficient	Wald Coefficient	*p*	Odds Ratio	Lower 95% Confidence Interval	Higher 95% Confidence Interval
Age	−0.207	1.708	0.191	0.813	0.596	1.109
Parity	0.586	0.179	0.672	1.797	0.119	7.080
Histology	−0.051	0.016	0.899	0.951	0.436	2.071
Stage	2.339	5.714	0.017	1.374	1.524	7.631
I Surgery type	0.053	0.002	0.967	1.055	0.083	3.373
II Surgery type	−1.689	0.036	0.689	0.019	0.002	3.681
Staging complete	−2.022	1.159	0.282	0.132	0.003	5.255
Chemotherapy I	−1.600	1.595	0.207	0.202	0.017	2.419
Constant	2.601	0.210	0.047	4.958		

**Table 4:** Regression model for NEOC survival after fertility-sparing surgery.

Parameters	B Coefficient	Wald Coefficient	*p*	Odds Ratio	Lower 95% Confidence Interval	Higher 95% Confidence Interval
Age	0.376	1.365	0.243	1.457	0.775	2.740
Parity	2.501	1.812	0.114	0.355	0.792	5.331
Histology	−0.537	0.339	0.560	0.584	0.096	3.564
Stage	−0.107	0.009	0.925	0.899	0.097	8.324
I Surgery type	−1.717	0.383	0.536	0.180	0.001	4.157
II Surgery type	−4.231	0.044	0.981	0.015	0.166	8.661
Staging complete	3.449	1.247	0.491	1.476	0.223	5.672
Chemotherapy I	1.200	0.165	0.684	3.321	0.010	6.245
Recurrence	−4.892	0.361	0.042	0.055	3.417	8.981
Chemotherapy II	2.777	0.015	0.866	2.620	0.012	4.371
Radicalization	1.434	0.277	0.599	4.197	0.020	4.275
Constant	9.557	0.421	0.028	3.905		

**Table 5:** Frequency of investigated pregnancy parameters and the differences between groups.

Parameters		Whole Sample		GCT		SCST		Between-Group *p*
		Number	Percent	Number	Percent	Number	Percent	
Pregnancy attempted	no	29	74.4	12	70.6	17	77.3	0.640
	yes	10	25.6	5	29.4	5	22.7	
Pregnancy achieved	no	3	30.0	2	40.0	1	20.0	0.513
	yes	7	70.0	3	60.0	4	80.0	
Delivery mode	vaginal	5	71.4	1	33.3	4	100	0.074
	caesarean section	2	28.6	2	66.7	0	0	
Delivery time	term	5	71.4	2	66.7	4	80.0	0.248
	preterm	2	28.6	1	33.3	1	20.0	

Legend: GCT—germ cell tumor, SCST—sex cord–stromal tumor.

## Data Availability

The data presented in this study are available from the corresponding author upon request due to the patient privacy regulations of our clinic.
